# Electroanatomic Mapping-Guided Radiofrequency Ablation of Adenosine Sensitive Incessant Focal Atrial Tachycardia Originating from the Non-Coronary Aortic Cusp in a Child

**DOI:** 10.1016/s0972-6292(16)30797-5

**Published:** 2014-10-06

**Authors:** Serhat Koca, Serkan Topaloglu, Serkan Cay, Aysenur Pac

**Affiliations:** 1 Department of Pediatric Cardiology, Yuksek Ihtisas Heart-Education and Research Hospital, Ankara, Turkey; 2 Department of Cardiology, Division of Arrhythmia and Electrophysiology, Yuksek Ihtisas Heart-Education and Research Hospital, Ankara, Turkey

**Keywords:** electroanatomic, focal, non-coronary cusp

## Abstract

Incessant focal atrial tachycardia may be encountered in the pediatric age group although it is rarely seen. Ablation using radiofrequency or cryothermal energy is the preferred method for drug-resistant cases. Recently, 3D electroanatomic mapping systems have been increasingly used for mapping and ablation. In this report, we presented, for the first time, a pediatric case with incessant focal atrial tachycardia originating from the non-coronary aortic sinus and ablated using 3D electroanatomic mapping system.

## Introduction

The majority of children presenting with paroxysmal supraventricular tachycardia (PSVT) have either accessory pathway mediated tachycardia or atrioventricular node reentry tachycardia. Atrial tachycardia is an uncommon type of PSVT in the pediatric age group [1,2]. Conventional methods and tools have been generally used for ablative purposes although recently, three-dimensional (3D) mapping systems have been increasingly used for mapping and ablation of arrhythmias in pediatric patients. Herein, we presented a pediatric case with atrial tachycardia mapped and ablated using 3D electroanatomic mapping system.

## Case report

**Figure 1:**
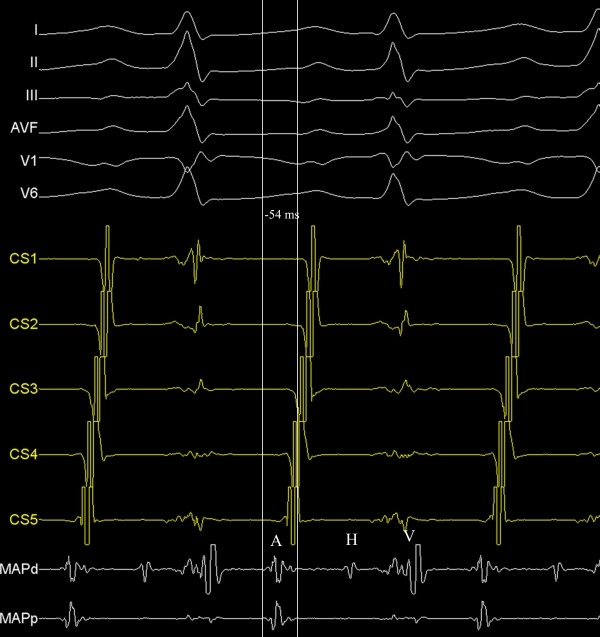
Intracardiac recordings showing the earliest atrial activation from the para-hisian region during tachycardia. A, atrial activation; CS, coronary sinus recording; H, His recording; MAP, ablation catheter recording; V, ventricular activation.

**Figure 2:**
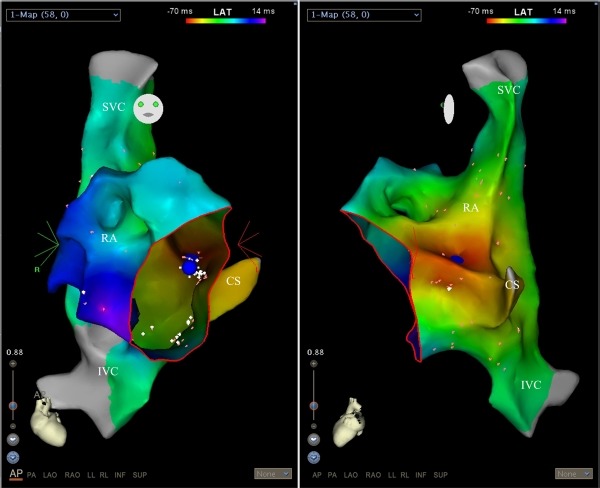
Carto 3 mapping showing the earliest atrial activation from the para-hisian region (blue dots) in postero-anterior (left panel) and steep left lateral (right panel) projections. CS, coronary sinus; IVC, inferior vena cava; RA, right atrium; SVC, superior vena cava. Red circle shows the tricuspid annulus.

**Figure 3:**
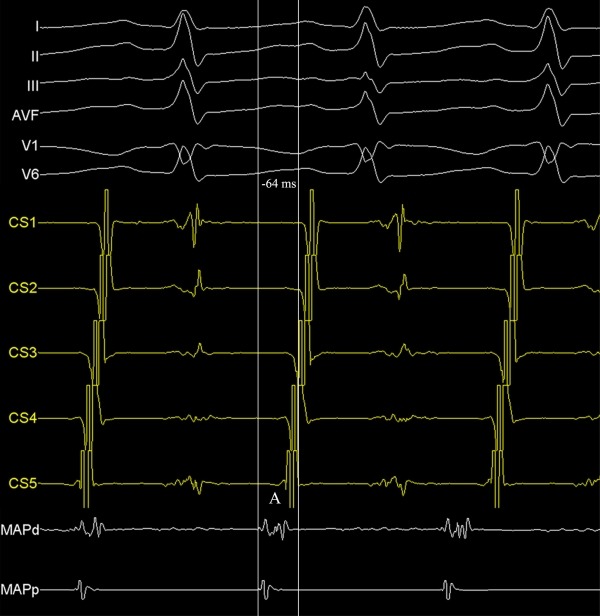
Intracardiac recordings showing the earliest atrial activation from the non-coronary cusp during tachycardia. A, atrial activation; CS, coronary sinus recording; MAP, ablation catheter recording. His and Ventricular recordings are not seen on MAP tracing.

**Figure 4:**
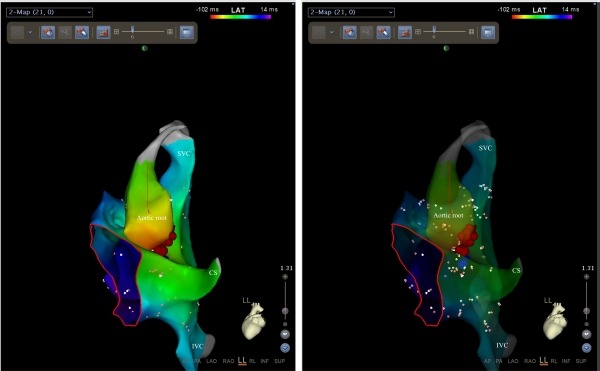
Carto 3 mapping showing the earliest atrial activation from the aortic root (non-coronary cusp) in the left lateral projection (left panel). Also, ablation points (red dots) are seen. In the right transparent panel, close anatomic relationship is seen between the para-hisian region and the non-coronary aortic cusp. CS, coronary sinus; IVC, inferior vena cava; SVC, superior vena cava. Red circle shows the tricuspid annulus.

An 8-year-old boy with recurrent and generally incessant palpitation attacks refractory to multiple antiarrhythmic drugs including beta-blockers, calcium channel blockers and amiodarone was referred to our center. Physical examination and echocardiography carried out on admission was unremarkable except the tachycardia. After application of adenosine, tachycardia stopped, but immediately repeated. The P-waves were positive in lead I and aVL, negative or indifferent in the inferior leads II, III, and aVF and biphasic in the precordial leads V1 and V2. For diagnostic and therapeutic purposes, electrophysiologic study was performed. First, right atrial map using fast anatomical mapping and local activation times (Carto®3 system, Biosense Webster, Belgium) during tachycardia was created. On 3D map, the earliest local activation times were detected from the para-hisian region. Local atrial activation preceded the onset of the P wave by 54 ms in this region (Figure 1 and 2). No ablation attempt was made and due to the close anatomic relationship with the non-coronary cusp, 3D map of the distal aorta using fast anatomical mapping and local activation times was also created. Local atrial activation preceded the onset of the P wave by 64 ms in this region (Figure 3). Combined 2 maps demonstrated very close anatomical relationship between the 2 different anatomical structures (Figure 4). Therefore, an earliest atrial activation preceded the atrial activation at the His bundle by 10 ms. The 3D propagation map also demonstrated tachycardia focus from the non-coronary cusp. Radiofrequency ablation from the non-coronary cusp using an irrigated tip catheter (ThermoCool® SF, Biosense Webster, Belgium) (20 W, 35 ºC and infusion rate of 15 ml/min) stopped the tachycardia immediately within 3 s. A second ablation attempt with higher power (30 W) due to recurrence permanently stopped the tachycardia. The safety burn was applied (Figure 4). Also, His potential was not detected at the successful site in the non-coronary cusp. No junctional beat and PR prolongation were observed during RF application. Re-evaluation after a waiting period of at least 30 minutes was performed and the arrhythmia could not be induced. The patient was asymptomatic during the 6-month follow-up.

## Discussion

To the best of our knowledge, this is the first child case with adenosine-sensitive focal atrial tachycardia originating from the non-coronary cusp, which was mapped and ablated using 3D electroanatomic mapping system.

In older children, spontaneous resolution or antiarrhythmic suppression of focal ATs are unlikely as in our case [3]. The first case with a focal AT and successfully ablated from the non-coronary aortic sinus has been described in 2004 [4]. Then similar publications have increased [5-7]. It has been demonstrated that focal atrial tachycardia originating from the septal regions are sensitive to adenosine in most patients [8,9]. In addition, Iwai et al. have demonstrated that most focal ATs terminated or transiently suppressed by adenosine although macroreentrant ATs were adenosine insensitive [10]. For patients with unsuccessful ablation attempts from the para-hisian region detailed electrophysiologic considerations including mapping from the non-coronary aortic sinus have been performed for successful ablation of the arrhythmia origin because the arrhythmia focus may have been located epicardially in the atrial wall. Radiofrequency ablation close to the His region carries a risk of inadvertent damage to the atrioventricular conduction system and can result in unsuccessful ablation attempts. In addition, mapping of the left side of the interatrial septum via transseptal route has inherent potential complications. Therefore, before energy application to the para-hisian region detailed mapping of neighboring non-coronary aortic cusp should be performed [6]. It should be kept in mind that retrograde approach from the non-coronary aortic sinus can be the first ablation strategy [7], especially in pediatric population.

Lastly, with the advent of 3D electroanatomic mapping system, ionizing radiation exposure to growing body with quite rapid cell cycle during mapping and ablation is substantially reduced.
